# Effects of a self-assembling peptide as a scaffold on bone formation in a defect

**DOI:** 10.1371/journal.pone.0190833

**Published:** 2018-01-05

**Authors:** Kei Ando, Shiro Imagama, Kazuyoshi Kobayashi, Kenyu Ito, Mikito Tsushima, Masayoshi Morozumi, Satoshi Tanaka, Masaaki Machino, Kyotaro Ota, Koji Nishida, Yoshihiro Nishida, Naoki Ishiguro

**Affiliations:** 1 Department of Orthopedic Surgery, Nagoya University Graduate School of Medicine, Showa-ku, Nagoya, Aichi, Japan; 2 Department of Ophthalmology, Osaka University Graduate School of Medicine, Suita, Osaka, Japan; University of South Carolina, UNITED STATES

## Abstract

Spinal fusion and bone defect after injuries, removal of bone tumors, and infections need to be repaired by implantation. In an aging society, recovery from these procedures is often difficult. In this study, we found that injection of SPG-178 leads to expression of several bone marker genes and mineralization in vitro, and revealed a significantly higher degree of newly formed bone matrix with use of SPG-178 in vivo. MC3T3-E1 cells were used to evaluate osteoblast differentiation promoted by SPG-178. To analyze gene expression, total RNA was isolated from MC3T3-E1 cells cultured for 7 and 14 days with control medium or SPG-178 medium. Among the several bone marker genes examined, SPG-178 significantly increased the mRNA levels for ALP, BMP-2 and Osteocalcin, OPN, BSP and for the Osterix. Ten-week-old female Wistar rats were used for all transplantation procedures. A PEEK cage was implanted into a bony defect (5 mm) within the left femoral mid-shaft, and stability was maintained by an external fixator. The PEEK cages were filled with either a SPG-178 hydrogel plus allogeneic bone chips (n = 4) or only allogeneic bone chips (n = 4). The rats were then kept for 56 days. Newly formed bone matrix was revealed inside the PEEK cage and there was an increased bone volume per total volume with the cage filled with SPG-178, compared to the control group. SPG-178 has potential in clinical applications because it has several benefits. These include its favorable bone conduction properties its ability to act as a support for various different cells and growth factors, its lack of infection risk compared with materials of animal origin such as ECM, and the ease with which it can be used to fill defects with complex shapes and combined with a wide range of other materials.

## Introduction

Spinal fusion and bone defect after injuries, removal of bone tumors, and infections need to repair by implantation. Usually, autografts with augmentation of metal devices have been performed because of their superior osteoinductivity and osteoconductivity. However, autograft bone is often limited in supply and its use has been found to be associated with donor site morbidity[1]. Allogeneic bone is another possible source of the materials. Although allograft bone may be obtained in greater quantities, the problems of complex processing, low biocompatibility and the risk of disease transfer also hinder its applications[2]. Therefore, tissue engineering products have emerged as an alternative approach to regenerate bone[3, 4].

The aims of using tissue engineering are to achieve the morphology and structure of the scaffold to increase adhesion of osteoblasts and osteoprogenitor cells, promote differentiation and migration, and allow synthesis of homogenous bone matrix without necrosis[5, 6]. There have been numerous reports on bone tissue engineering using β-tricalcium phosphate (β-TCP), a bioactive ceramic material with good properties of resorption, osteoconductivity, cellular adhesion, mechanical strength, and compatibility with host bone tissue[7–9]; and hydroxyapatite (HA), a calcium phosphate crystal that constitutes the inorganic component of native bone and exhibits good osteoinduction and biocompatibility[10, 11]. However, these materials are solids and cannot penetrate between chip bones.

Self-assembling peptides may be candidate materials to solve these problems. These peptides are administered in a hydrogel form and have advantages of injectability, filling space between grafted bones, and excellent biocompatibility[12]. The complete sequence of a self-assembling peptide was originally found in a region of alternating hydrophobic and hydrophilic residues in zuotin[13], which is characterized by a stable b-sheet structure that undergoes self-assembly into nanofibers. The nanofibers form interwoven matrices that further form a hydrogel scaffold[14, 15]. These hydrogel systems are well characterized and have already been employed in a variety of tissue engineering studies [16–19], and drug delivery systems[20]. Self-assembling peptides are a 100% chemically synthesized material. Therefore, the use of self-assembling peptide hydrogels can minimize the risk of biological contamination and the influence from the undefined factors.

We developed a self-assembling peptide, SPG-178 (Self-assembling Peptide Gel, amino acid sequence #178; [CH_3_CONH]-RLDLRLALRLDLR-[CONH_2_]; R = arginine, L = leucine, D = aspartic acid, A = alanine), as a scaffold and potential therapeutic agent for SCI[21]. The stability of the peptide solution/hydrogel at neutral pH (the isoelectric point, at which a protein has a zero net charge and reaches minimum solubility) contributes to the biocompatibility of the scaffold and provides an additional benefit for the sterilization procedure [22]. The concern with regard to the clinical use of hydrogel is its mechanical strength. With regard to this concern, based on the idea that it should be possible to develop ideal bone filler materials by combining the strength of artificial bone with the bone regeneration and bone conduction properties of hydrogel, we used a hybrid scaffold system that involves fabricating a cage from polyetheretherketone (PEEK), a material that is used in clinical applications such as spinal fusion procedures[23–25], filling the interior of this cage with hydrogel according to the previous report[25].

In this study, we show increased expression of bone marker genes in SPG-178-promoted osteogenesis in vitro, and improved bone healing with use of SPG-178 in vivo. These results provide new evidence for the role of SPG-178 as a scaffold for osteogenesis through induction of osteoconductive factors.

## Materials and methods

### Cell seeding

MC3T3-E1 cells (RCB1126, an osteoblast-like cell line from C57BL/6 mouse calvaria) were obtained from the RIKEN Cell Bank (Tsukuba, Japan). MC3T3-E1 cells were cultured at 37°C in 5% CO_2_ atmosphere in α-modified minimal essential medium (α-MEM; GIBCO). Unless otherwise specified, the medium contained 10% heat-inactivated fetal bovine serum (FBS), 100 U/mL penicillin, and 100 mg/mL streptomycin.

### Cell culture

Ninety-six-well chamber slides (Nunc, Roskilde, Denmark) were coated with SPG-178 (30 μl) (0.8% w/v hydrogel; Menicon Co.) and left one hour at 37°C, and the medium contained 10% FBS over night at 5°C. The MC3T3-E1 cells were then digested in pre-warmed 0.05% trypsin (Invitrogen, USA) at 37°C for 5 min in a conical flask agitated by hand every 5 min. After digestion, the supernatant was removed and the remained trypsin was inactivated with Dulbecco’s modified Eagle’s medium (DMEM; Invitrogen) supplemented with 20% heat-inactivated FBS (Invitrogen) at room temperature (RT). Afterwards, cells were centrifuged for 3 min at 1,500 rpm. The supernatant was removed and the cells were resuspended in serum free medium, and added SPG-178 (1: 1). 20μl cells with hydrogel were plated onto culture plates coated with SPG-178 removed the supernatant at a density of 100000 cells/well on each dish for 1 hour at 37°C. At last 200ml DMEM containing 20% heat-inactivated FBS were added. As controls, chamber slides with the same number of cells were prepared. The next day, the cells undergo differentiation and mineralization when cultured in differentiating medium containing 50 mg/mL ascorbic acid and 3.0 mM b-glycerophosphate[26].

### Real-time reverse transcription-polymerase chain reaction (RT-PCR)

To analyze gene expression, we isolated total RNA from MC3T3-E1 cells cultured for 7 and 14 days with 2 types of medium: control group medium and SPG-178 group medium. mRNA expression levels were determined for bone marker genes: alkaline phosphatase (ALP), bone morphogenetic proteins-2 (BMP-2), osteocalcin (OC), osteopontin (OPN), bone sialoprotein (BSP), and osterix. Quantitative RT-PCR analysis of total RNA was performed on cells extracted with TRIzol reagent (Invitrogen) and purified with RNeasy columns (Qiagen, Valencia, CA, USA). Expression levels of selected mRNAs were quantified using real-time RT-PCR. Differences in expression between groups were expressed using cycle time (Ct) values as relative increases. With the control as 100%, and assuming that the Ct value reflects the initial copy number and there was a 100% efficacy, a difference of one cycle is equivalent to a twofold difference in the copy number. Sequences of primers used for RT-PCR are listed in Table 1. All the PCR reactions were performed at least in triplicate and the expression levels were normalized to glyceraldehyde-3-phosphate dehydrogenase (GAPDH) signal in the same reaction. Primer sequences and product sizes are shown in Table 1.

**Table 1:** Primer sequences used in quantitative RT-PCR.

	Forward	Reverse
GAPDH	GGCTCTGCTACTACCGATGC	GGCTTGTTTAGGCTCCTCCT
ALP	CTTGACTGTGGTTACTGCTGATCA	GTATCCACCGAATGTGAAAACGT
Osteocalcin	GCTGCCCTAAAGCCAAACTCT	AGAGGACAGGGAGGATCAAGTTC
BMP-2	TGACTGGATCGTGGCACCTC	CAGAGTCTGCACTATGGCATGGTTA
Osteopontin	TGGTGGTGATCTAGTGGTGCCAA	CACCGGGAGGGAGGAGGCAA
Osterix	TCAGCCGCCCCGATCTTCCA	AATGGGTCCACCGCGCCAAG
BSP	AGACCAGGAGGCGGAGGCAG	TTGGGCAGTTGGAGTGCCGC

### Fabrication of hybrid scaffolds

The PEEK cages were made by Yasojima Proceed Co. Ltd, (Kobe, Japan), using TECAPEEK CLASSIX (Ensinger, Nufringen, Germany). These cages were tubular structures of outer diameter 5 mm, inner diameter 3 mm, and height 5 mm. Four elliptical holes were formed in the side wall of these cylinders.

### Animals

**Fig 1:**
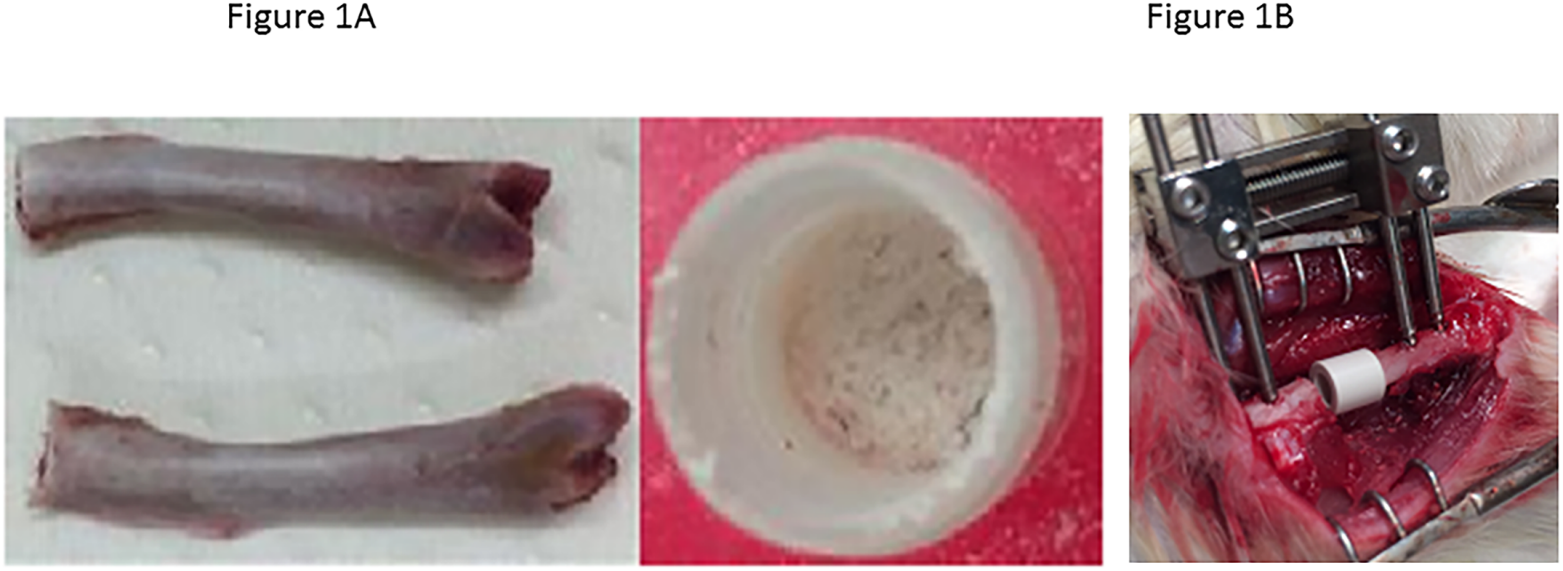
Bone defect model. (A) For allogeneic bone, femurs of other rats were smashed the status to pieces. (B) The bony defect was either left intact or filled with a prepared scaffold and compressed by external fixation.

Eight female Wistar rats, ten-week-old and 300–350 g (Nippon CLEA, Tokyo, Japan), were used for all transplantation procedures. Animal experiments were performed in strict accordance with the Guide for the Care and Use of Laboratory Animals (National Research Council, 1996) and all efforts were made to minimize suffering. All animal procedures were approved by the Institutional Animal Care and Use Committee of Nagoya University for the use of laboratory animals. For allogeneic bone, femurs of other rats were smashed the status to pieces (Fig 1A).

### Bone defect model

The rats were anesthetized with sodium pentobarbital (40 mg/kg). Perioperative anesthesia was maintained by inhalation of isoflurane. Under sterile conditions, a posterolateral incision was made in the left femur, and the thigh muscles were divided. The midshaft of the femur was exposed and fixed using an external fixator and four pins of diameter 1.4 mm (Meira, Gifu, Japan), and a 5-mm-long section of diaphysis was then removed together with the periosteum using a microcutting saw (IMPLATEX, Tokyo, Japan). The bony defect was filled with a prepared scaffold (SPG-178 and allogeneic chip bone) (Fig 1A) and compressed by external fixation (Fig 1B). The wounds were closed with size 5–0 nylon sutures. A PEEK cage was implanted into a bony defect (5 mm) within the left femoral mid-shaft, and stability was maintained by an external fixator. The PEEK cages were filled with either a SPG-178 hydrogel (200μl) plus allogeneic bone chips (0.2g, n = 4) or only allogeneic bone chips (0.2g, n = 4). The rats were then kept for 56 days. All animals were given antibiotics in their drinking water [1.0 ml of Bactramin (Roche) in 500 ml of acidified water] for 2 weeks after surgery. An injection of meloxicam (0.1–0.2mg/kg body weight), an anti-inflammatory analgesic, was administered intramuscularly for 3 days postoperatively. We performed animal monitoring twice a day for 1 week after surgery, and then once a day until sacrifice. Decrease in eating, drinking and moving or clear suffering from pain were determined to be the humane end points and the animals would have been terminated immediately if these signs were exhibited. At 56 days after surgery, animals were anesthetized with pentobarbital sodium (40 mg/kg) and perfused through the heart with 100 ml saline and then 250 ml of 4% paraformaldehyde in phosphate buffer (pH 7.4).

### Quantitative micro-CT analysis of bone repair in response to cell seeded scaffolds

Intact femurs were first imaged using digital X-ray (SOFTEX, CMB-2, Kanagawa, Japan) before trimming and scanning with the Skyscan 176 micro-CT (after removal of pins and side plate). Samples were scanned at an energy of 55 kVp and intensity of 145 mA with 226 ms integration time, resulting in an isotropic voxel size of 36 μm. From the scanned volume, a cylindrical region of interest (ROI), corresponding to the defect size of 5 mm diameter and at the location of the original defect, was selected for analysis. After segmentation of the mineralized tissue with a threshold of 220 (equivalent to 312 mg hydroxyapatite/ cm^3^ (mgHA/cm^3^)), a Gauss filter width of 0.8, and filter support of 1.0, the mineralized matrix volume was quantified throughout the entire construct and pre-sented as bone volume in mm^3^. The bone volume per total volume (BV/TV) ratio was calculated as previously described[27]. Representative cross-sections and longitudinal sections were cut out after 3D reconstruction with the built-in software of the micro-CT.

### Histological analysis of bone repair in response to cell seeded scaffolds

Un-decalcified bones were embedded at low temperature in PMMA and serial 5 μm sections were stained with 5% silver nitrate (Von Kossa) and counterstained with 0.2% toluidine blue to distinguish mineral from soft tissue (Alizarin Red). Adjacent sections were stained with Naphthol AS-TR phosphate (Sigma-Aldrich) in Tris-maleate buffer pH 9.3 to identify alkaline phosphatase (ALP) activity in osteoblasts.

### Statistical analysis

Statistical analysis was performed using SPSS (SPSS Inc., Chicago, IL, USA), using an unpaired two-tailed Student t test for single comparisons. In all analyses, significance was accepted at p < 0.05.

## Results

### In vitro assays

**Fig 2:**
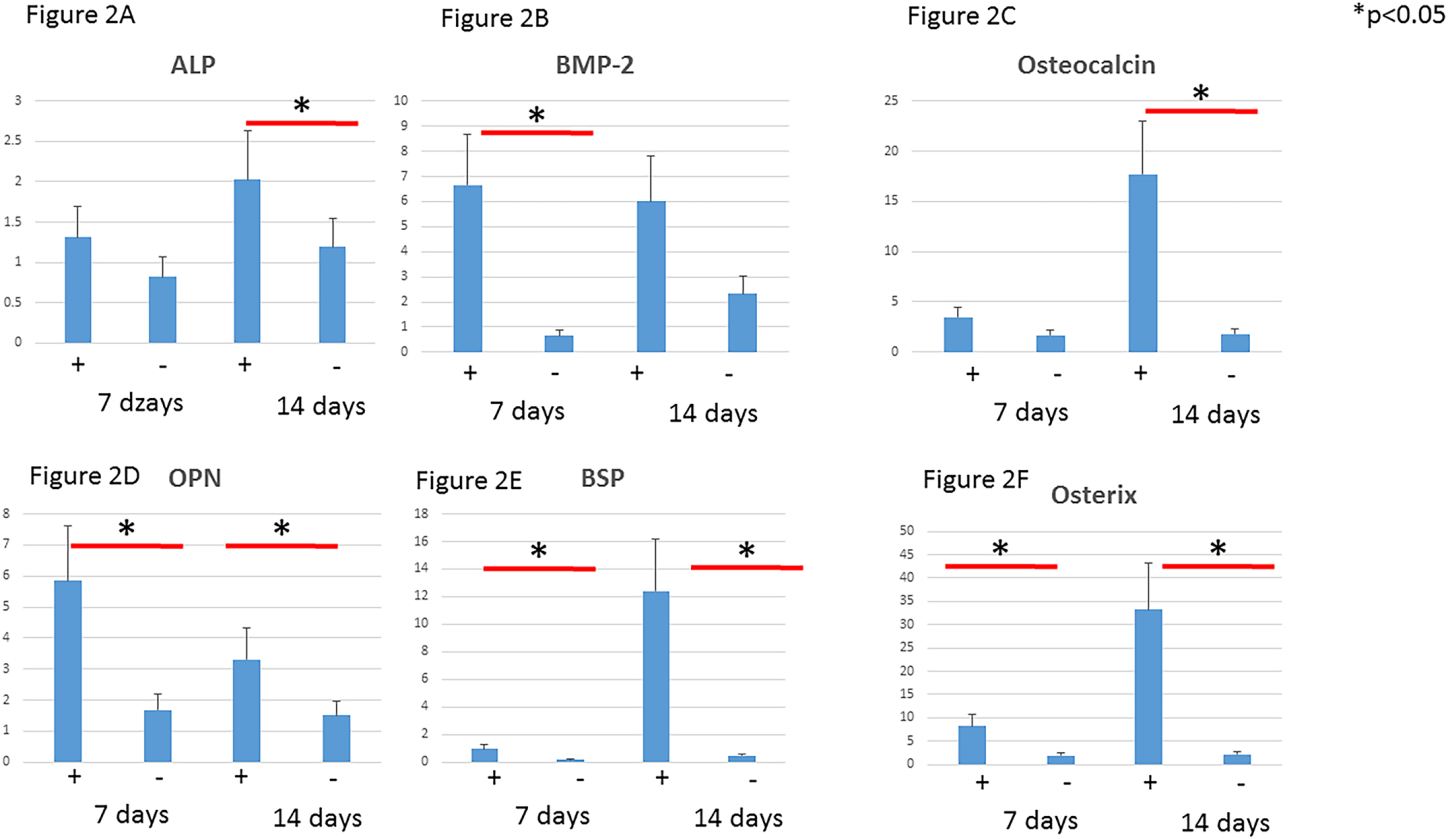
Real-time reverse transcription-polymerase chain reaction. (A) ALP. (B) BMP-2. (C) Osteocalcin. (D) Osteopontin. (E) BSP. (F) Osterix.

We first examined the effects of SPG-178 on osteoblast differentiation of MC3T3-E1 cells. Real-time PCR was used to quantify expression as the effect of SPG-178 on mRNA expression of several bone marker genes (Fig 2). In the SPG-178 group, levels of mRNAs for ALP (14 days), BMP-2 (7 days) and Osteocalcin (14 days), OPN (7 and 14 days), BSP (7 and 14 days) and for the Osterix (7 and 14 days), were significantly increased compared to the control group (p<0.05) (Fig 2A–2D).

### In vivo experiments

#### Radiological findings

**Fig 3:**
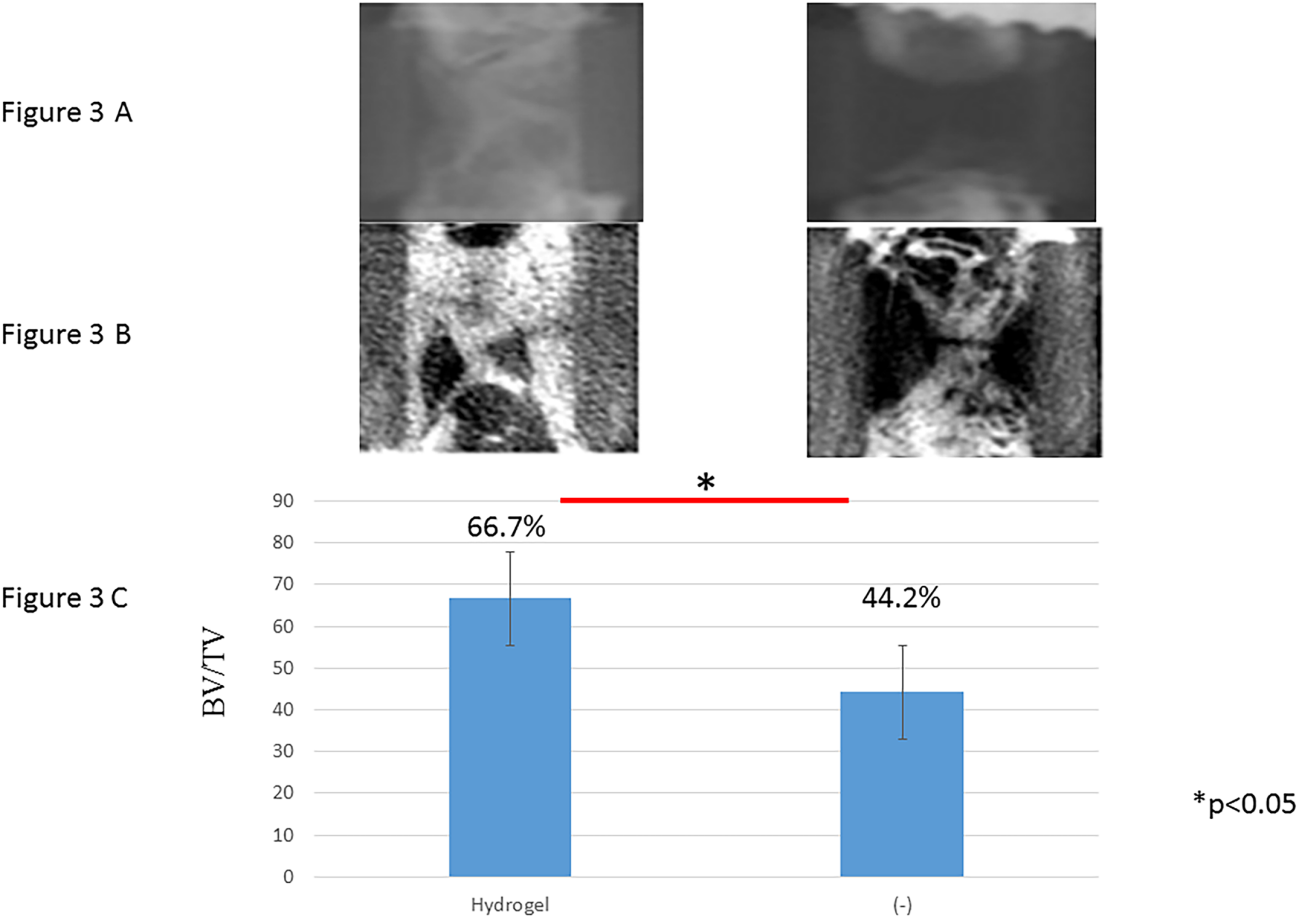
X-ray and quantitative micro-CT analysis of bone repair in response to cell seeded scaffolds. (A) X-ray. (B) CT. (C) Quantitative micro-CT analysis.

The bone formation capacity of scaffolds with different modifications and different patterns was assessed in vivo after transplantation in a rat femoral defect. Transplants were recovered after 56 days, subjected to X-ray and micro-CT analysis (Fig 3A and 3B). Micro-CT analysis revealed a significantly higher degree of newly formed bone matrix in all experimental groups compared to the negative control group (chip bone only) (Fig 3C). The empty defects remained primarily devoid of any mineralized tissue throughout the study, showing that they were of critical size (non-healing within the length of the study).

#### Histology

**Fig 4:**
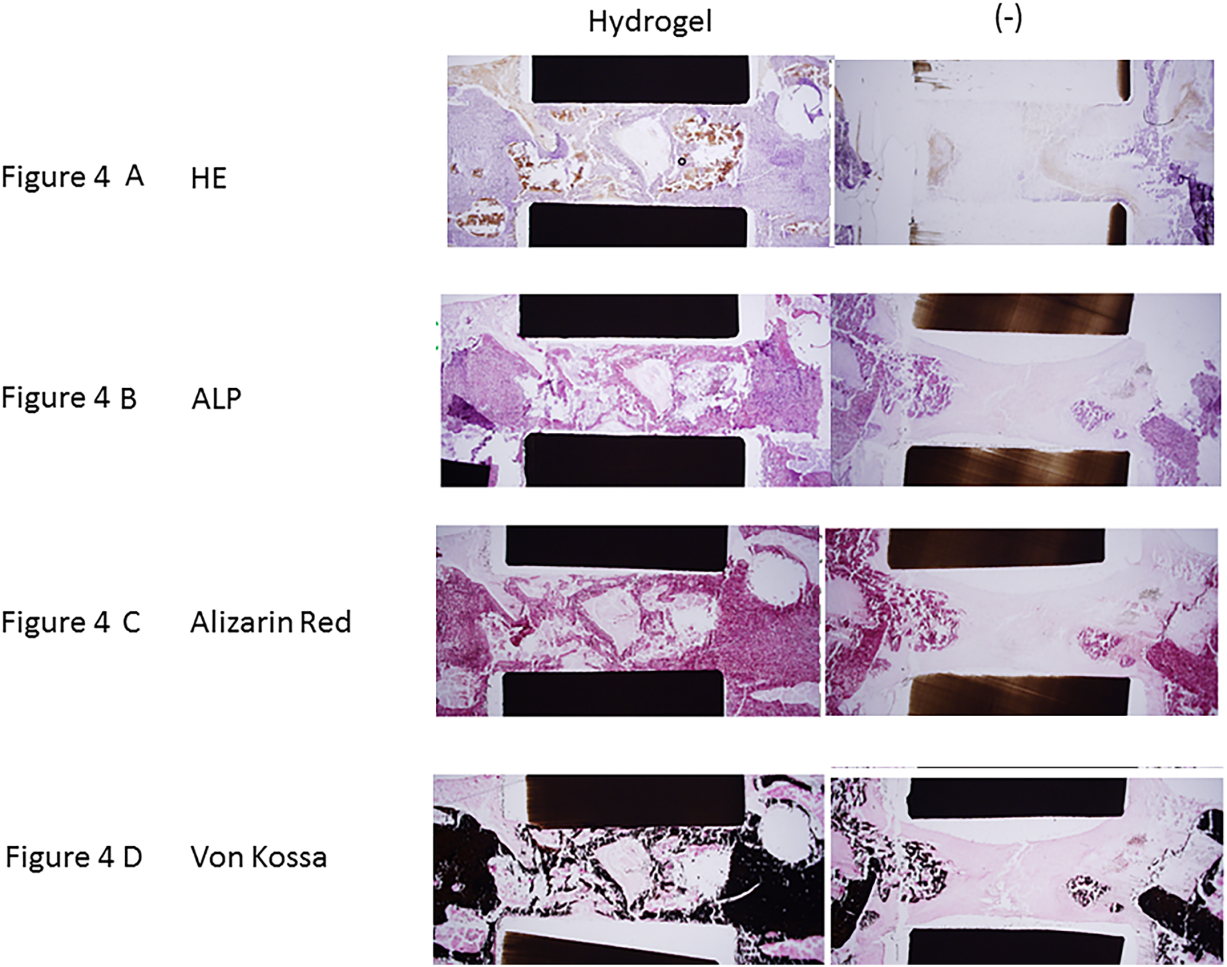
Histological analysis of bone repair in response to cell seeded scaffolds. (A) HE. (B) ALP. (C) Alizarin red. (D) Von Kossa.

Representative H&E staining demonstrated clear defect bridging in empty group and a good bonding from the host bone in hydrogel groups (Fig 4A). Alkaline phosphatase (ALP) shows significant ALP activity in osteoblasts (Fig 4B). Alizarin red and Von Kossa staining result showed that Hydrogel group was higher amount of calcium compared to control group (Fig 4C and 4D).

## Discussion

The factors necessary for bone formation include cells such as osteoblasts, an extracellular matrix to support the adhesion and movement of these cells, and various intercellular functions such as storage of materials, a supply of nutrients (e.g., by angiogenesis) necessary for tissue formation, cytokines to promote and control cell growth and dynamic factors such as strength and stability[28, 29]. Although recombinant human BMP-2 (rhBMP-2) has been used in several surgical applications, the high dosage of BMP-2 used in surgery compared with the endogenous concentration may result in complications associated with pleiotropic BMP-2 diffusion from the intended site, including ectopic bone formation, immunological reactions, and tumourigenesis[30, 31]. The ideal graft substitute should reabsorb with time to allow and promote new bone formation while maintaining its properties as an osteoconductive scaffold until it is no longer required. β-TCP has advantages over HA when used as a filler, in that it is more rapidly reabsorbed[32]. Peptides are administered as hydrogels, which is advantageous compared to β-TCP and HA, which are used as solids, in that the peptide hydrogel is injectable, can fill space between grafted bones, and has excellent biocompatibility[12]. The generated nanofibres mimic the natural extracellular matrix (ECM) and enhance attachment, growth and differentiation of a variety of cells, including chondrocytes and osteoblasts[33, 34] A self-assembling peptide gel, RADA16, has been used in tissue engineering studies[16–19]. However, this hydrogel has a very low pH (approximately 3–4) and is unstable under neutral conditions, thereby retaining the potential to harm inner cells and host tissues[22]. SPG-178 peptide solution (2.4 mM) is transparent and able to form a stable hydrogel at neutral pH[22]. Several studies have examined SPG-178 as a scaffold[21, 22, 35], with increased expression of osteopontin, osteocalcin and collagen type I found in dental pulp stem cells cultured in SPG-178 gel with osteogenic induction medium[35].

In this study, we found that injection of SPG-178 leads to expression of several bone marker genes and mineralization. The SPG-178 peptide solution (2.4 mM) is transparent and able to form a stable hydrogel at neutral pH when triggered by an increase in salt concentration. The stability of the peptide solution/hydrogel at neutral pH contributes to the biocompatibility of the scaffold. The solution can also be sterilized with an autoclave, which is advantageous in the sterilization procedure. It not only works as a support for cells and growth factors, but has also been reported to be highly conductive for the vascular system[36]. However, the drawbacks of hydrogel as scaffold in the process of bone regeneration need its strength and stability.

In the hybrid scaffold we used from a PEEK cage filled with hydrogel, the PEEK cage makes up for the shortfall in the strength of hydrogel, and is thus able to satisfy all the above-mentioned requirements for bone formation. Furthermore, there have hitherto been no proposals of the concept where separate materials are used for parts responsible for regeneration and parts responsible for conferring strength, such as by using a PEEK cage for strength and hydrogel for bone regeneration and bone conduction. This method may make it possible to develop the ideal bone filler[25].

Among the several bone marker genes examined, SPG-178 significantly increased the mRNA levels for ALP (14 days), BMP-2 (7 and 14 days) and Osteocalcin (14 days), OPN (7 days), BSP (14 days) and for the Osterix (14 days) (Fig 2A–2F). It is commonly accepted that osteogenic differentiation is controlled by a variety of bone-related markers, such as osterix, ALP, OC, BSP, and OPN. These markers are induced differently according to the stages of differentiation. OPN, OC, and BSP are mainly expressed from the middle to the late phases of differentiation[37, 38]. It was considered that those bone markers that osteoblasts activated by SPG-178 hydrogel exerted promoted mineralization, and this hydrogel is suitable as a scaffold. The charged amino acid residues within the peptide nanofiber, especially the positively charged arginine residues, are considered to support cell adhesion at the beginning of the culture[22, 39]. The serum proteins in the cell culture medium may also attach to the peptide nanofiber and help the cell adhesion[40]. The charge interactions of the positively charged polymer (amines) to the negatively charged membrane potential can improve cell attachment and mobilization.

In control group (chip bone), we only observed slight bone formation at the cut ends of the bone because the PEEK cage on its own has no bone conduction abilities. On the other hand, in hydrogel group where the cage was filled with SPG-178, we obtained good bone formation inside the PEEK cage and increased BV/TV (Figs 3 and 4). The fact that there was no SPG-178 hydrogel at the 56 days post grafting time point indicates the hydrogel become incorporated by replacement resorption and little physical space exists for new bone growth.

Although the structure of hydrogel is fragile, by filling the interior of a sufficiently strong PEEK cage with hydrogel and forming a gel, it seems that it was possible to maintain the structure even in the bone defect parts, and to achieve good bone conduction performance. It also seems that cells such as vascular endothelial cells, bone marrow mesenchymal stem cells, and osteoblasts penetrate into hydrogel from an early stage, allowing angiogenesis and osteogenesis to proceed[36].

## Conclusions

SPG-178 has potential in clinical applications because it has several benefits. These include its favorable bone conduction properties its ability to act as a support for various different cells and growth factors, its lack of infection risk compared with materials of animal origin such as ECM, and the ease with which it can be used to fill defects with complex shapes and combined with a wide range of other materials.
